# Effect of household toilet accessibility on physical health of ethnic minority adolescents: a longitudinal study from the China Education Panel Survey 2013 and 2014

**DOI:** 10.1186/s12889-023-15547-5

**Published:** 2023-04-12

**Authors:** Yao Jiang, Fan Yang

**Affiliations:** 1 Department of Demography, Zhou Enlai School of Government, Nankai University, Tianjin, China; 2 Department of Labor and Social Security, School of Public Administration, Sichuan University, Chengdu, China

**Keywords:** Toilet accessibility, Ethnic minority adolescents, Sanitation infrastructure, Health rights

## Abstract

**Background:**

Accessing household toilets is vital for adolescent health during crucial stages of growth and development; however, some ethnic minority families in China lack toilets. The relationship between household toilet accessibility and the physical health of adolescents in China’s ethnic minority areas has been underexamined.

**Methods:**

Using two waves of data from the China Education Panel Survey (2013 and 2014), this study explored the effect of household toilet accessibility on Chinese ethnic minority adolescents’ physical health. The sample consisted of 576 ethnic minority adolescents with an average age of approximately 13 years. To test the health effect of household toilet accessibility on ethnic minority adolescents, a fixed effects ordinary least squares model and difference-in-differences combined with propensity score matching method were used.

**Results:**

The results of the fixed effects model and difference-in-differences combined with propensity score matching showed a significant and positive effect of household toilet accessibility on adolescents’ physical health. The heterogeneity analysis indicated that among the toilet types of soil cesspits, cement cesspits, squat toilets, and flush toilets, the flush toilets were proven to be the most efficient in improving adolescents’ physical health. Moreover, the family living arrangement was proven to play a moderating role in the effect of household toilet accessibility on the physical health of ethnic minority adolescents. The average marginal effects of household toilet accessibility on the physical health of ethnic minority adolescents who lived without parents were more evident and significant than those who lived with their parents.

**Conclusion:**

Promoting household toilet accessibility in China’s ethnic minority areas is essential for improving adolescents’ health. Moreover, having a household toilet is not sufficient, the quality and dignity of household toilets are also important.

## Background

Accessing toilets is crucial for public health and providing toilets has been referred to as a barometer of civilization [1, 2]. Promoting access to toilets can prevent human fecal pollution, decrease pathogen exposure, and promote good health outcomes in adolescents during growth and development [3]. However, due to traditional cultural practices and constrained financial resources, some ethnic minority families in China still lack toilets [4]. Furthermore, the relationship between household toilet accessibility and the health of adolescents in China’s ethnic minority areas has been underexamined in the existing literature. Using two waves of data from the China Education Panel Survey (CEPS), this study explored the effect of household toilet accessibility on Chinese ethnic minority adolescents’ physical health.

There is a wealth of research on the effects of toilet sanitation facilities on the health of adolescents worldwide, and many have found that toilet accessibility is closely linked to adolescents’ health outcomes [5–10]. For instance, a case study from Bengaluru, India, revealed that adolescent girls who lack access to toilets have to use open defecation, which makes them prone to reproductive tract infections, urinary tract infections, and snake bites [5]. In addition, a study conducted in India using school-going adolescents as a sample group indicated that access to toilets could significantly reduce incidences of diarrheal [6]. According to empirical research conducted in Venezuela, a lack of toilet accessibility could increase sexual violence and negatively affect the physical and psychological health of young female adolescents [7]. A qualitative study conducted in the United States found that restricted toilet use might significantly affect bladder health among adolescents [8].

Furthermore, some studies have revealed that the quality and dignity of toilets also have an important effect on adolescents’ health [9, 10]. Specifically, a study of 13-to-17-year-old students in Bahia, Brazil, using semi-structured interviews, argued that toilet acceptability, quality, and safety were also essential for adolescents to avoid the risk of diarrhea [9]. According to a Danish empirical study that recruited adolescents aged 11.2 years on average, poor-quality toilets were significantly correlated with students’ toilet refusal and bladder and bowel dysfunction symptoms [10]. However, studies on the effect of household toilet accessibility on the physical health of adolescents in China’s ethnic minority groups are relatively sparse.

The toilet facilities of Chinese families are undergoing changes and development. In the 1960s, the Chinese government initiated a patriotic public health campaign in an effort to minimize infectious diseases and improve health conditions. This campaign involved encouraging people to use toilets and wash their hands afterwards [4]. In the 1980s, the Chinese government promoted the “Three Improvements” project, which included improvements to toilet facilities, water quality, and health education in rural areas [4]. Moreover, the “Toilet Revolution” has become a hot phrase since 2015 [11]. Since then, it has been substantially implemented in China as a campaign to ensure the hygienic separation of human excreta and environmental pollution from human contact, and to realize resource recycling [12].

However, implementing toilet improvement projects and the “Toilet Revolution” in China’s ethnic minority areas has been difficult. For example, in 2017, the prevalence of sanitary toilets in the rural areas of the Ningxia Hui and Xinjiang Uyghur Autonomous Regions was 74.3% and 65.6%, respectively, which was much lower than the average prevalence of sanitary toilets in the country (81.8%) [13]. The local natural environment, economic conditions, and people’s opinion on toilets all contribute to the low prevalence of sanitary toilets in ethnic minority communities. The inhabited areas of China’s ethnic minorities are typically remote both environmentally and economically. The natural environment is harsh, and economic development is relatively slow [14, 15]. In China’s ethnic minority areas, implementing toilet improvement projects faces not only the technical challenges of climate (e.g., freezing temperatures or drought), but also the financial constraints of maintaining the normal functioning of toilet sanitation facilities [4].

From the perspective of public opinion, each ethnic group has its own toilet civilization with a distinct and lengthy history of fecal disposal and toilet use. Consequently, cultural clashes among ethnic groups frequently occur during toilet improvement projects. For instance, the Yi ethnic people, who respect fire, tend to oppose the promotion of household toilets with biogas digesters [16]. They regard biogas fires that are produced using human and animal feces as filthy, and they will not use these for heat and cooking [16]. In addition, Tibetans clearly distinguish between inside and outside items [4]. Outside items are filthy, and, to a certain extent, feces from the human body belong outside [17]. Therefore, they are hesitant to build toilets in their houses and generally lack them [4]. Overall, toilet improvement in China’s ethnic minority areas is not only a vital public health issue, but also a profound social and cultural movement that embodies the contradiction between modern public health concepts and traditional perspectives [18]. In the existing literature, however, few studies have attempted to explore the effect of household toilet accessibility on the health of adolescents in China’s ethnic minority areas.

Thus, this study’s contributions are as follows: First, using two waves of CEPS data (2013 and 2014), we investigated the effect of household toilet accessibility on the physical health of ethnic minority adolescents in China (including but not limited to Mongolian, Tibetan, Yi, Manchu, Hui, Zhuang, and Uyghurs), which constitutes a group worthy of attention but relatively ignored in previous studies. Second, we determined the heterogeneous health effects of different toilets (including soil cesspit, cement cesspit, squat toilet, and flush toilet) on ethnic minority adolescents. Third, we examined the moderating role played by different family living arrangements (i.e., adolescents who live with parents and those who live without parents) in the effect of household toilet accessibility on ethnic minority adolescents’ physical health. The findings of this study have policy implications for the promotion of the “Toilet Revolution” and provide guidelines for the healthy development of adolescents in China’s ethnic minority areas and other developing countries and regions.

## Methods

### Data

Data of this study were obtained from the CEPS, which was conducted by the National Survey Research Center at the Renmin University of China. The CEPS is a nationally representative longitudinal survey with two waves (i.e., CEPS2013 and CEPS2014) that examines the influences of family, school, community, and society on the development of Chinese adolescents. The first wave of CEPS2013 was conducted anonymously and confidentially during 2013–2014, with a follow-up investigation during 2014–2015 [19]. Using a stratified, multistage sampling design with a probability proportionate to size, the CEPS randomly selected a school-based, nationally representative sample of seventh to ninth graders and their parents nested in 28 county-level units in China [20]. In the Chinese educational regime, the seventh to ninth graders are generally secondary school adolescents between 13 and 15 years of age. Thus, the CEPS provides an appropriate dataset to explore the effect of household toilet accessibility on adolescents’ physical health.

We cleaned the data by excluding missing items, outliers, and other abnormal values. Furthermore, since our focus was the effect of household toilet accessibility on the physical health of ethnic minority adolescents, respondents of the Han ethnic group were excluded, as Han is China’s majority nationality. Finally, 576 ethnic minority seventh to ninth graders were matched across the CEPS2013 and CEPS2014. Approximately 46% of the ethnic minority students in our sample were male, and the mean age of the samples was 13.477 years old.

### Measures

#### Dependent variables

In this study, the dependent variable was the physical health of ethnic minority adolescents. This was measured by asking the parents of these adolescents, “How would you rate your child’s physical health?” On a five-point Likert scale, the response of “very unhealthy” was coded as “1”, “somewhat unhealthy” as “2,” “normal” as “3,” “somewhat healthy” as “4,” and “very healthy” as “5.”

To test the robustness of the empirical results, we used the adolescents’ self-rated physical health and height as alternative dependent variables. The variable of adolescents’ self-rated physical health was acquired by asking these adolescents, “How would you rate your current physical health?” On a five-point Likert scale, the answer of “very unhealthy” was coded as “1,” “somewhat unhealthy” as “2,” “normal” as “3,” “somewhat healthy” as “4,” and “very healthy” as “5.” In general, adolescents’ body height is closely linked with their physical health [21]. Thus, adolescents’ height during the survey year was also employed as an alternative dependent variable.

#### Independent variables

The toilet accessibility was a binary independent variable in this study. These ethnic minority adolescents were asked, “Does your house have a toilet?” The answer of “Yes” was coded as “1”; otherwise, “0” was used.

To capture the heterogeneous health effects of different forms of toilet accessibility on ethnic minority adolescents, we obtained information on toilet type by further asking these adolescents whose houses have toilets: “What kind of toilets do you have in your house?” The responses to the question were categorized as follows: (1) soil cesspit, (2) cement cesspit, (3) squat toilet, and (4) flush toilet. In the empirical analysis, the toilet type was treated as a categorical variable.

#### Moderating variable

The family living arrangement is a vital factor in the improvement of family facilities and adolescents’ physical health [22, 23]. In some economically underdeveloped ethnic minority areas, the young labor force largely flows out to seek jobs, resulting in intergenerational co-residence and left-behind children [24]. Compared to adolescents who reside with their parents, adolescents who do not reside with their parents may receive less family health education and healthcare intervention, and may therefore have worse physical health. Thus, to test the moderating role of the family living arrangement in the effect of household toilet accessibility on the health of ethnic minority adolescents, we classified the family living arrangement into two categories: (1) living with parents and (2) living without parents (including living alone and living with grandparents, relatives, and others). Living with parents was coded as “1”; otherwise, it was coded as “0.”

#### Control variables

To precisely determine the effect of household toilet accessibility on ethnic minority adolescents’ physical health, we controlled for the variables that could affect their physical health. First, family economic conditions may be prerequisites for adolescents to acquire healthy foods and better medical services [25–31]. Thus, we controlled the three variables of family financial condition, family income level, and parents’ occupations. Second, the living environment is an important external factor that may affect the physical health of ethnic minority adolescents [32–34]. Thus, we controlled for environmental sanitation and environmental pollution around adolescents’ living houses. In addition, we considered the health effects of safe water and controlled for the variable of tap water. Third, adolescents’ lifestyle behaviors were considered [35]. Although we did not find questions about adolescents’ lifestyle behaviors in the CEPS, we found a variable that may indirectly reflect adolescents’ lifestyle behaviors—close friends’ smoking and drinking behaviors. Due to the influence of peers, adolescents’ lifestyle choices may be impacted by close friends’ smoking and drinking habits to a large extent [35, 36]. We thus controlled for adolescents’ close friends’ behaviors of smoking and drinking. Table 1 presents the definitions and descriptive analyses of these variables.

**Table 1:** Variable definition and descriptive analysis

Variables	Definition	Mean	S.D	Min	Max
Explained variable					
Adolescents’ physical health	1 = very unhealthy; 2 = somewhat unhealthy; 3 = normal; 4 = somewhat healthy; 5 = very healthy	4.071	0.931	1	5
Adolescents’ self-rated physical health		3.842	0.950	1	5
Adolescents’ height	Adolescents’ body height in survey year (cm)	160.221	8.314	130	175
Explanatory variable					
Toilet accessibility	1 = the house has a toilet = 1; 0 = otherwise	0.751	0.433	0	1
Toilet type	1 = soil cesspit; 2 = cement cesspit; 3 = squat toilet; 4 = flush toilet	–	–	–	–
Control variable					
Family financial condition	1 = very difficult; 2 = somewhat difficult; 3 = normal; 4 = somewhat good; 5 = very good	2.585	0.676	1	5
Family income level	1 = very low; 2 = somewhat low; 3 = normal; 4 = somewhat high; 5 = very high	2.503	0.735	1	5
Parents’ occupations	1 = at least one parent of an adolescent is engaged in non-agricultural work; 0 = otherwise	0.497	0.500	0	1
Environmental sanitation	The environmental sanitation around the house of adolescent is: 1 = very bad; 2 = somewhat bad; 3 = somewhat good; 4 = very good	2.735	0.801	1	4
Environmental pollution	The environmental pollution around the house of adolescent is: 1 = seriously polluted; 2 = somewhat polluted; 3 = slightly polluted; 4 = non-polluted	2.030	0.850	1	4
Close friends’ behaviors of smoking and drinking	Among adolescents’ five close friends, 1 = none of them smoking and drinking; 2 = one or two of them smoking and drinking; 3 = three or more of them smoking and drinking	1.113	0.353	1	3
Tap water	1 = the house of adolescent has tap water; 0 = otherwise	0.867	0.340	0	1
Moderating variable					
Family living arrangement	1 = living with parents; 0 = otherwise	0.761	0.426	0	1
Observations	1152				

### Statistical analyses

#### Fixed effects ordinary least squares model

As the key dependent variable of this study was ethnic minority adolescents’ physical health, measured on a five-point Likert scale, adolescents’ physical health can be considered continuous data. The results of the Hausman test (Chi^2^ = 32.010, *p* < 0.01) rejected the null hypothesis of a random effect. Consequently, we investigated the effect of household toilet accessibility on ethnic minority adolescents’ physical health by estimating the following fixed effects ordinary least squares (OLS) model:

1 $${Adolescent{s}^{^{\prime}}physical health}_{it}={\beta }_{0}+{\beta }_{1}{Toilet accessibility}_{it}+\sum {y}_{j}{Z}_{jit}+{\mu }_{i}+{year}_{t}+{\sigma }_{c}+{\varepsilon }_{it}$$

where the dependent variable is ${Adolescent{s}^{^{\prime}}physical health}_{it}$, it represents the physical health of ethnic minority adolescents. $\text{Toilet }{\text{accessibility}}$ is a binary indicator and represents the independent variable. ${\beta }_{0}$ is the intercept, and ${\beta }_{1}$ is the coefficient for having a household toilet. ${Z}_{jit}$ indicates the *j* time-varying control variable that may affect ethnic minority adolescents’ physical health. ${\mu }_{i}$ and ${\text{year}}_{\text{t}}$ are the fixed effects of individual and year, respectively. ${\upsigma }_{\mathrm{c}}$ is for the fixed effect of counties where the adolescents lived, and ${\varepsilon }_{it}$ is the error term. Equivalent to a dummy variable for each ethnic minority adolescent, ${\mu }_{i}$ captures all time-constant characteristics of an adolescent, even when these characteristics are unobservant. With ${year}_{t}$ and ${\upsigma }_{\mathrm{c}}$ as the equivalent of the dummy variables for each survey year and the living county of adolescents, the fixed effects model further accounts for any year-to-year and county-to-county changes that may affect adolescents’ physical health [37].

#### Difference-in-differences combined with propensity score matching method

Using a fixed effects model, we obtained the effect of household toilet accessibility on the physical health of ethnic minority adolescents without the influences of individual time-invariant factors and year-to-year and county-to-county shifts to some extent. To capture the “true” health effect of household toilet accessibility on adolescents, we need to compare the health outcomes of adolescents whose houses have toilets with those of adolescents whose houses do not have toilets. However, there may be systematic differences among these adolescents. If we compare the two groups of respondents directly, the estimation results may be biased owing to systematic differences in the samples. Consequently, we face the challenge of a lack of counterfactual information. The difference-in-differences (DID) combined with propensity score matching (PSM) method is the preferred method for constructing a counterfactual framework and precisely obtaining the health effect of household toilet accessibility on ethnic minority adolescents [38, 39].

To construct an appropriate control group (respondent’s house does not have a toilet) and compare outcomes to those of the treatment group (respondent’s house has a toilet), we used the PSM estimation based on the propensity scores. With the PSM estimation, we can identify individuals in the control group with similar characteristics as those in the treatment group and exclude observations that were not on common support [39]. In the analysis, the physical health of adolescents was considered a continuous variable. Consequently, based on the observations of common support of the PSM estimation, the following equation of the DID model was derived for the continuous outcomes of adolescents’ physical health:

2 $${Y}_{it}={\beta }_{1}+{\beta }_{2}{ Toilet accessibility}_{it}+{\beta }_{3}{After}_{t}+{\delta Toilet accessibility}_{it}\times {After}_{t}+{yX}_{jit}+{year}_{t}+{\varepsilon }_{it}$$

where ${\text{Y}}_{\text{it}}$ denotes the physical health status of ethnic minority adolescents *i* in year *t*. ${\text{Toilet accessibility}}_{\text{it}}$ is a binary indicator and represents a dummy variable. ${\text{Toilet accessibility}}_{\text{it}}$ = 1 represents the treatment group in which the house of ethnic minority adolescents had toilets, whereas ${\text{Toilet accessibility}}_{\text{it}}$ = 0 indicates the control group in which the house of ethnic minority adolescents lacked toilets. ${\text{After}}_{\text{t}}$ represents a time dummy variable. ${\text{After}}_{\text{t}}$ = 0 means 2013, and ${\text{After}}_{\text{t}}$ = 1 represents 2014. ${Toilet accessibility}_{it}\times {After}_{t}$. is the interaction between groups and time. ${\text{X}}_{\text{jit}}$ is the *j* covariate of ethnic minority adolescent *i* at time *t*. ${\text{year}}_{\text{t}}$ indicates the fixed effect of the survey year. ${\varepsilon }_{it}$ means error term. $\delta$ denotes the average treatment effect, also known as the DID value. $\delta$ is the coefficient in the DID model in which we are most interested.

## Results

### Results of fixed effects ordinary least squares model

The results of the fixed effects OLS model are presented in Table 2. As shown in Table 2, toilet accessibility was significantly and positively associated with ethnic minority adolescents’ physical health $\left(\beta =0.306, p<0.01\right)$ when control variables were omitted from the model. Furthermore, after controlling for control variables and the fixed effects of individual, year, and county, toilet accessibility remained significantly and positively correlated with ethnic minority adolescents’ physical health $\left(\beta =0.337, p<0.01\right)$. These results indicated that compared with adolescents whose families did not have toilets, adolescents whose families had toilets had better physical health.

**Table 2:** Results of fixed effects ordinary least squares regression model

Variables	Adolescents’ physical health	
Toilet accessibility	0.306***	0.337***
	(0.096)	(0.097)
Family financial condition		-0.046
		(0.068)
Family income level		0.016
		(0.061)
Parents’ occupations		1.051***
		(0.352)
Environmental sanitation		0.093**
		(0.045)
Environmental pollution		0.090*
		(0.047)
Close friends’ behaviors of smoking and drinking		-0.026
		(0.093)
Tap water		0.003
		(0.115)
Family living arrangement		0.278***
		(0.107)
Constant	4.645***	2.917***
	(0.502)	(0.660)
Fixed effects		
Individual	Yes	Yes
Year	Yes	Yes
County	Yes	Yes
R^2^	0.721	0.733
Observations	1152	

^a^ Standard errors are in the parentheses

^b^ ***, **, * Significance levels at 1, 5, and 10%, respectively

Table 2 shows that the control variables of parents’ occupations $\left(y=1.051, p<0.01\right)$, environmental sanitation $\left(y=1.093, p<0.05\right)$ and environmental pollution $\left(y=0.090, p<0.1\right)$ around adolescents’ place of residence, and family living arrangement $\left(y=0.278, p<0.01\right)$ had significant and positive influences on ethnic minority adolescents’ physical health when utilizing the fixed effects OLS model.

### Heterogeneous effect of different toilet types

Table 3 presents, using the fixed effects OLS model, the heterogeneous effects of different toilet types (including soil cesspit, cement cesspit, squat toilet, and flush toilet) on the physical health of ethnic minority adolescents.

**Table 3:** Heterogeneous effect of different toilet types (fixed effects ordinary least squares model)

Variables	Adolescents’ physical health	
Soil cesspit as reference		
Cement cesspit	0.607***	0.552***
	(0.204)	(0.207)
Squat toilet	0.901***	0.833***
	(0.190)	(0.196)
Flush toilet	1.229***	1.162***
	(0.225)	(0.233)
Family financial condition		-0.130
		(0.082)
Family income level		0.262
		(0.353)
Parents’ occupations		0.116
		(0.074)
Environmental sanitation		0.061
		(0.056)
Environmental pollution		0.027
		(0.054)
Close friends’ behaviors of smoking and drinking		-0.081
		(0.105)
Tap water		-0.267
		(0.192)
Family living arrangement		0.171
		(0.116)
Constant	3.717***	3.489***
	(0.492)	(0.679)
Fixed effects		
Individual	Yes	Yes
Year	Yes	Yes
County	Yes	Yes
R^2^	0.709	0.712
Observations	760	

^a^Standard errors are in the parentheses

^b^*** Significance level at 1%

^c^Observations included in heterogeneity analysis are adolescents whose houses have toilets in both 2013 and 2014

Accounting for the control variables and the fixed effects of individual, year, and county, the coefficients of cement cesspit $\left(\beta =0.552, p<0.01\right)$, squat toilet $\left(\beta =0.833, p<0.01\right)$, and flush toilet $\left(\beta =1.162, p<0.01\right)$ were positive and significant. Among the three toilet types, the coefficient of the flush toilet was the highest, whereas that of the cement cesspit was the lowest. Overall, in comparison to the soil cesspit, the three types of toilets (i.e., cement cesspit, squat toilet, and flush toilet) can improve ethnic minority adolescents’ physical health more efficiently. Moreover, the flush toilet had the most positive effect on health, followed by the squat toilet, and the soil cesspit had the worst effect.

### Moderating effect of family living arrangement

**Fig. 1:**
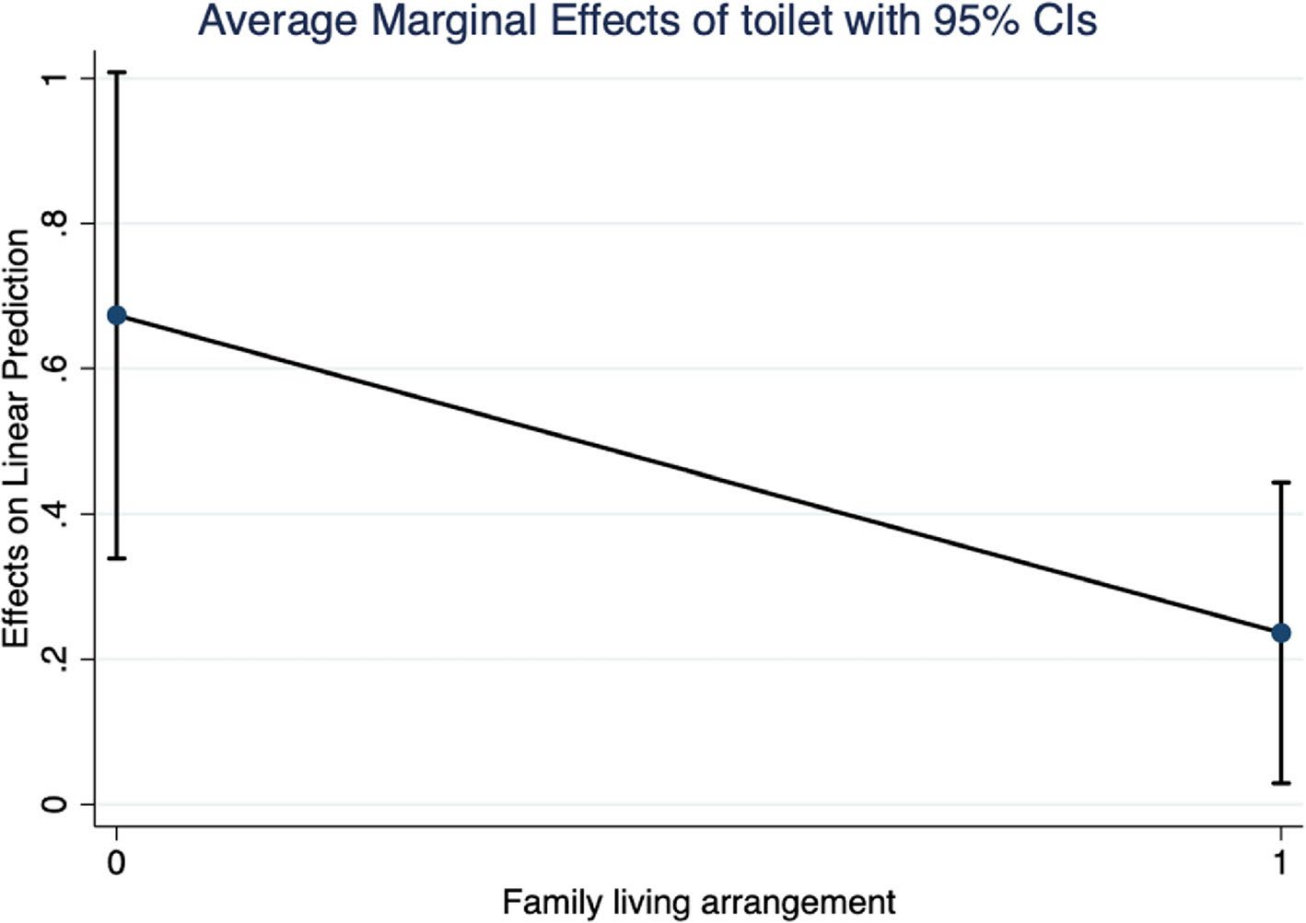
Moderating effect of family living arrangement**.** ^a^ The results are estimated by the fixed effects (individual, year, and county) ordinary least squares model added the interaction term of toilet accessibility and family living arrangement. ^b^ “1” means the adolescents living with parents, and “0” indicates the adolescents living with grandparents, relatives, and other. ^c^ CI means confidence interval. ^d^ The 95% confidential interval of adolescents who live with parents is [0.029 – 0.443], and the average marginal effect is 0.236. The 95% confidential interval of the adolescents who live with grandparents, relatives, and others is [0.334 – 1.008], and the average marginal effect is 0.673

Figure 1 shows the average marginal effect of household toilet accessibility on the physical health of adolescents from different family living arrangements. Figure 1 shows that the 95% confidential interval (CI) of adolescents who live with their parents was [0.029 – 0.443], and the average marginal effect was 0.236. Moreover, the 95% CI of the adolescents who live without parents was [0.334 – 1.008], and the average marginal effect was 0.673. These results reveal that the average marginal effects of household toilet accessibility on the physical health of ethnic minority adolescents who lived with grandparents, relatives, and others were more evident and significant than those who lived with their parents.

### Results of difference-in-differences combined with propensity score matching

The estimation results for DID combined with PSM are presented in Table 4. Using the three matching algorithms: nearest neighbor matching $\delta =0.335, p<0.01$, radius matching $\delta =0.335, p<0.01$, and kernel matching $\delta =0.335, p<0.01$, the results of DID combined with PSM implied that the household toilet accessibility could significantly improve ethnic minority adolescents’ physical health. These results are consistent with those estimated using the fixed effects model.

**Table 4:** Results of difference-in-differences combined with propensity score matching by employing three matching algorithms

Variable	Adolescents’ physical health		
	Nearest neighbor	Radius	Kernel
$\mathrm{Toilet accessibility}\times \mathrm{After }(\mathrm{DID value})$	0.335***	0.335***	0.335***
	(0.121)	(0.121)	(0.121)
Toilet accessibility	0.440***	0.440***	0.440***
	(0.077)	(0.077)	(0.077)
After	0.775***	0.775***	0.775***
	(0.093)	(0.093)	(0.093)
Constant	3.744***	3.744***	3.744***
	(0.054)	(0.054)	(0.054)
Control variables	Yes	Yes	Yes
R^2^	0.087	0.087	0.087
Observations	1152		

^a^Standard errors are in the parentheses

^b^*** Significance level at 1%

^c^The coefficient of Toilet accessibility $\times$ After is $\delta$ (i.e., DID value), the coefficient of Toilet denotes ${\beta }_{2}$, and the coefficient of After is ${\beta }_{3}$ in Eq. (2)

^d^The element number of the nearest-neighbor matching with a caliper was 1, the radius was set to 0.01 in radius matching, and kernel matching used default kernels and bandwidth

Table 5 provides a comparison of the samples after PSM. Except for the key outcome variable of adolescents’ physical health, the *p* values of other variables’ mean differences between the control and treatment groups were greater than 10% after applying the PSM estimation. Hence, after matching the control and treatment groups, the findings indicated that, except for the key outcome variable, there were no significant differences among the variables, and the DID combined with the PSM model was correctly specified.

**Table 5:** Comparison between control and treatment groups after employing propensity score matching

Variable	Mean		Difference	T statistic	*p*-value
	Control	Treatment			
Adolescents’ physical health	3.744	4.184	0.440	5.56	0.000***
Family financial condition	2.709	2.721	0.012	0.23	0.818
Family income level	2.685	2.675	-0.009	0.17	0.863
Parents’ occupations	0.534	0.566	0.032	0.77	0.444
Environmental sanitation	2.680	2.788	0.107	1.38	0.167
Environmental pollution	2.340	2.239	-0.103	1.26	0.207
Close friends’ behaviors of smoking and drinking	1.098	1.076	-0.021	0.73	0.463
Tap water	0.907	0.914	0.007	0.28	0.778
Family living arrangement	0.815	0.761	-0.053	1.56	0.120
Observations	1152				

^a^ *** Significance level at 1%

### Robustness checks

To test the robustness of the results, subgroup regressions were conducted, the estimation method was changed, and the dependent variable was replaced. First, we considered the gender of ethnic minority adolescents and their parents’ occupations. The full sample was categorized into boys and girls based on gender. The ethnic minority adolescents were divided into two subgroups according to their parents’ occupations: non-agricultural and other. Table 6 provides the different subgroups regression results using the fixed effects OLS models. When the control variables and fixed effects were controlled for, as indicated in Table 6, the toilet accessibility was significantly and positively related to the physical health of ethnic minority adolescents in the subgroups of boys $\left(\beta =0.333, p<0.05\right)$ and girls $\left(\beta =0.366, p<0.01\right)$, and subgroups of parents with non-agricultural occupations $\left(\beta =0.480, p<0.01\right)$ and other $\left(\beta =0.261, p<0.05\right)$. Thus, these results are consistent with those estimated by using the entire sample.

**Table 6:** Subgroup regression (fixed effects ordinary least squares model)

Variable	Adolescents’ physical health			
	Boy	Girl	Non-agricultural	Other
Toilet accessibility	0.333**	0.366***	0.480***	0.261**
	(0.138)	(0.140)	(0.141)	(0.130)
Family financial condition	-0.066	-0.047	0.022	-0.030
	(0.100)	(0.093)	(0.092)	(0.097)
Family income level	0.095	-0.006	0.003	0.004
	(0.088)	(0.088)	(0.087)	(0.084)
Parents’ occupations	0.254	2.261***	–	–
	(0.440)	(0.580)	–	–
Environmental sanitation	0.122*	0.045	-0.001	0.158**
	(0.067)	(0.062)	(0.064)	(0.062)
Environmental pollution	0.060	0.090	0.085	0.093
	(0.072)	(0.063)	(0.062)	(0.068)
Close friends’ behaviors of smoking and drinking	0.051	-0.179	-0.140	0.013
	(0.117)	(0.156)	(0.115)	(0.141)
Tap water	-0.142	0.102	-0.273	0.063
	(0.175)	(0.159)	(0.199)	(0.145)
Family living arrangement	0.325**	0.164	0.180	0.385**
	(0.148)	(0.163)	(0.137)	(0.156)
Constant	3.525***	2.184**	4.412***	1.005
	(0.753)	(0.854)	(0.621)	(0.693)
Fixed effects				
Individual	Yes	Yes	Yes	Yes
Year	Yes	Yes	Yes	Yes
County	Yes	Yes	Yes	Yes
R^2^	0.757	0.740	0.768	0.739
Observations	530	622	572	580

^a^Standard errors are in the parentheses

^b^***, **, * Significance levels at 1, 5, and 10%, respectively

Second, because the dependent variable of ethnic minority adolescents’ physical health on a five-point Likert scale can also be viewed as ordered and discrete data, we used an alternative estimation method of the fixed effects ordered probit model to confirm the effect of household toilet accessibility on ethnic minority adolescents’ physical health. Table 7 displays the results of the fixed effects ordered probit model. These results demonstrated that household toilet accessibility continued to have a significant and positive effect on adolescents’ physical health $\left(\beta =0.911, p<0.01\right)$.

**Table 7:** Results of ordered probit model and using alternative dependent variable (fixed effects)

Variable	Adolescents’ physical health	Adolescents’ self-rated physical health	Adolescents’ height
	Ordered probit	OLS	OLS
Toilet accessibility	0.911***	0.258***	1.896***
	(0.190)	(0.100)	(0.638)
Family financial condition	-0.160	-0.049	0.010
	(0.137)	(0.069)	(0.445)
Family income level	0.038	-0.083	0.141
	(0.121)	(0.063)	(0.402)
Parents’ occupations	2.244***	1.904***	-0.163
	(0.618)	(0.361)	(2.315)
Environmental sanitation	0.273***	0.102**	0.224
	(0.091)	(0.046)	(0.297)
Environmental pollution	0.263***	0.049	0.208
	(0.096)	(0.048)	(0.307)
Close friends’ behaviors of smoking and drinking	-0.082	-0.183*	-0.918
	(0.176)	(0.095)	(0.609)
Tap water	-0.051	-0.241**	-1.246*
	(0.212)	(0.118)	(0.755)
Family living arrangement	0.803***	0.336***	0.055
	(0.219)	(0.110)	(0.705)
Constant	–	3.043***	166.858***
	–	(0.677)	(4.338)
Fixed effects			
Individual	Yes	Yes	Yes
Year	Yes	Yes	Yes
County	Yes	Yes	Yes
Pseudo R^2^	0.512	–	–
R^2^	–	0.731	0.856
Observations	1152		

^a^Standard errors are in the parentheses

^b^OLS means the ordinary least squares model

^c^***, **, * Significance levels at 1, 5, and 10%, respectively

Third, the dependent variable of adolescents’ physical health was replaced with their self-rated physical health and body height. Table 7 shows the results of the robustness check for substituting the dependent variable using the fixed effects OLS model. The results indicated that household toilet could have a significant and positive effect on adolescents’ self-rated physical health $\left(\beta =0.258, p<0.01\right)$ and body height $\left(\beta =1.896, p<0.01\right)$, suggesting that the positive effect of household toilet accessibility on ethnic minority adolescents’ physical health was valid across different methods and models.

## Discussion

The toilet is one of the most significant factors in expanding the human lifespan [40]. A previous study has argued that the contribution of modern sanitary facilities to public health is more important than that of antibiotics, vaccines, or anesthesia [4]. Adolescents are at a crucial stage of growth and development, and the surrounding sanitary environment has a profound effect on their health. The effect of household toilet accessibility on the physical health of adolescents in China’s ethnic minority areas has been underfocused and underexamined in the existing literature. Using two waves of data from the CEPS, we examined the effect of household toilet accessibility on the physical health of adolescents from Chinese ethnic minority groups. We found that household toilet accessibility had a significantly positive effect on the physical health of ethnic minority adolescents.

Our results are similar to those of previous studies that focused on the influences of family or school sanitary facilities on the health of adolescents in South Asia, South America, and Europe [5–10]. Although these conclusions are consistent, the toilet accessibilities in China’s ethnic minority areas cannot be improved instantaneously. There is a blind spot in technological rationality; namely, the civilization and history, which is often the internal logic behind the failure of modern technology projects that aim to improve people’s quality of life [16]. When focusing on toilet issues in China’s ethnic minority areas, the implementation of the “Toilet Revolution” policy and related projects should be founded on a complete understanding and respect for the civilization and history of the particular ethnic group. For instance, local government can use successful toilet improvement cases to publicize the health benefits of using household toilets and gradually guide people to adopt more hygienic public health practices. Moreover, the government can try to rely on the local elites in ethnic minority areas as role models to motivate people’s enthusiasm for toilet improvements.

To promote ethnic minority adolescents’ physical health more comprehensively, it is not sufficient to simply provide household toilets; the quality and dignity of household toilets are also crucial. Compared with the soil cesspit, the toilet types of cement cesspit, squat toilet, and flush toilet could more effectively improve ethnic minority adolescents’ physical health, especially the flush toilet. However, the harsh climatic and environmental conditions of ethnic minority areas are not conducive to the use of flush toilets, and climatic and environmental barriers are difficult to control. This may explain why there are fewer flush toilets in these ethnic minority areas. To more efficiently improve ethnic minority adolescents’ physical health, alternatives for flush toilets should be widely developed. For example, central and local governments could proactively promote environment-friendly toilets (such as vacuum toilets and water-free toilets) for households in ethnic minority areas, rather than limiting the usage of these environment-friendly toilets to scenic spots [41]. Such toilets can work just as well as flushing toilets and simultaneously have the advantages of saving water, reducing noxious odors, and effective disposal [42]. Meanwhile, local governments need to provide households in ethnic minority areas with a method of installing environment-friendly toilets and establish a long-term maintenance system for these toilets by utilizing market mechanisms.

Moreover, in the “Toilet Revolution”, ethnic minority adolescents living alone and with grandparents, relatives, and others should receive more attention than those living with their parents. In the analysis of the moderating effect of the family living arrangement, we discovered that the physical health of adolescents living without parents was more likely to be significantly affected by household toilet accessibility than the health of those living with their parents. China has over six million left-behind children under the age of 16 whose parents have left rural areas for urban employment in cities [43, 44]. Of these, 96% lived with their grandparents [44]. To a certain extent, the health and lifestyles of most adolescents living without parents may be influenced by their grandparents, whom themselves have a poor understanding of health and hygiene. Therefore, if the household sanitary facilities of ethnic minority adolescents who live without parents can be improved and their school health education is strengthened, their physical health may be significantly improved.

We also found that among the regression coefficients of the control variables, the regression coefficient of parents’ occupations was large and significant. This implies that parents’ occupations have a significant influence on children’s physical health. Compared to parents who are farmers or unemployed, parents who are engaged in non-agricultural work may directly improve the family economic and provide better opportunities for children to obtain healthcare services. Therefore, it is so economically beneficial for parents to have non-agricultural jobs to promote the physical health of their children.

Furthermore, in the robustness regression that used a gendered split sample, the variable of parents’ occupations was very significant for girls’ physical health, but not for boys. China has a historical tradition of prioritizing boys over girls; however, this situation has gradually disappeared with the improvement in parents’ educational levels and economic conditions [45]. Thus, if adolescents’ parents are engaged in non-agricultural jobs, they may have higher educational levels and better economic conditions than parents who are farmers or unemployed, and their children, especially girls, can be given better access to health services and have good physical health.

This study had some limitations that deserve mention. First, each ethnic group has its own toilet civilization; therefore, we recommend improvements in future research. Focusing on a specific ethnic minority group can reveal the history and development of household sanitary facilities in greater depth and provide more practical policy implications. Thus, future studies should investigate the effect of household toilet accessibility on adolescents’ physical health from the perspective of a particular ethnic group. Second, restricted by the data availability, we utilized adolescents’ close friends’ lifestyle behavior as an alternative variable for adolescents’ lifestyle behavior based on peer influence. If the data are available, future studies should directly control for variables related to adolescents’ lifestyle behaviors.

## Conclusions

Employing two waves of nationally representative data from the CEPS, this study explored the effect of household toilet accessibility on Chinese ethnic minority adolescents’ physical health. By using the fixed effects OLS model and DID combined with the PSM method, we found that there was a statistically significant and positive impact of household toilet accessibility on Chinese ethnic minority adolescents’ physical health. Compared with soil cesspits, the toilet types of cement cesspit, squat toilet, and flush toilet could improve adolescents’ physical health substantially, and the flush toilets could improve adolescents’ physical health the most. Moreover, the family living arrangement played a moderating role in the effect of household toilet accessibility on adolescents’ physical health. The physical health of ethnic minority adolescents who lived without their parents was more evidently and significantly affected by household toilet accessibility than that of those who lived with their parents. Therefore, improving household toilet accessibility in China’s ethnic minority areas is essential for promoting adolescents’ health.

## Data Availability

The study utilizes secondary sources of data that are freely available in the public domain through http://www.cnsda.org/index.php?r=projects/view&id=61662993. Those who wish to access the data may register at the above link and thereafter can download the required data free of cost.

## Abbreviations

CEPS: China Education Panel Survey
OLS: Ordinary least squares
DID: Difference-in-differences
PSM: Propensity score matching
CI: Confidential interval
